# Implementation of a Golden Hour protocol for initial management of preterm infants: a quality improvement study

**DOI:** 10.3389/fped.2026.1832031

**Published:** 2026-05-20

**Authors:** Sarah Dénes, Estelle Stockis, Vincent Rigo, Sophie Tribolet

**Affiliations:** 1 Neonatology Division, University Hospital of Liège, Belgium; 2 Neonatology Unit, Citadelle Hospital, Liège, Belgium

**Keywords:** golden hour, neonate, preterm birth, prospective study, standardization

## Abstract

**Purpose:**

This study implemented and evaluated a systematic approach for managing preterm infants <31 weeks of gestational age (wGA) and/or estimated fetal weight (EFW)<1300 g during their first hour of life. The primary focus was organizational efficiency, thermal regulation, and hypoglycemia prevention, with secondary assessment of clinical outcomes and team perception.

**Methods:**

A prospective observational study was conducted from January 2022 to July 2023 in a tertiary neonatal intensive care unit (NICU). Infants meeting inclusion criteria were compared with a retrospective 2019 control cohort. The Golden Hour (GH) protocol emphasized anticipation, role assignment, and early interventions, including umbilical venous catheterization (UVC), and parenteral nutrition (PN) initiation. Outcomes included procedural metrics (e.g., time to incubator closure, UVC placement) and clinical outcomes (e.g., hypothermia, hypoglycemia, mortality, and major comorbidities of prematurity). Team perception was assessed via survey.

**Results:**

77 infants were included in the GH group and 72 infants in a retrospective control group. GH infants experienced less hypothermia (23/77 vs. 36/72 -*p* = 0.03) and first blood glycemia evaluation was significantly earlier [43 (35–50) vs. 63 (53–77) min -*p* < 0.001]. The median incubator closure time was 64 (58–71) min, close to the GH target of 60 min. Rates of hypoglycemia, mortality, and major comorbidities of prematurity (e.g., bronchopulmonary dysplasia, necrotizing enterocolitis) were similar between groups. Team members reported improved anticipation, communication, and job satisfaction following protocol implementation.

**Conclusion:**

The GH protocol improved thermal regulation and team efficiency without altering clinical outcomes. Its positive effect on teamwork supports further evaluation in larger studies.

## 1 Introduction

Premature infants are highly vulnerable, and their stabilization immediately after birth is complex (1, 2). During the transition to extrauterine life, they experience respiratory, circulatory, metabolic, and thermal challenges, compounded by the sudden interruption of placental nutrient supply. In adult trauma care, the “Golden hour” concept emphasizes the importance of rapid, well-coordinated interventions to improve outcomes (3). Reynolds et al. (4) first applied this concept to neonatal medicine. In neonatology, the Golden Hour (GH) refers to the first 60 minutes of life (MOL), during which structured, timely, evidence-based interventions can influence morbidity and stabilization quality. Several studies and meta-analysis (5) found that standardized GH protocols improve thermal stability, glycemic control, and process efficiency while enhancing teamwork and communication.

In our neonatal unit, clinical observations and informal staff feedback suggested variability in the management of preterm infants during the first hour of life. Differences were particularly apparent in thermal regulation, respiratory support, and early glucose monitoring–domains known to influence early neonatal outcomes. This variability highlighted the need to improve reliability, consistency, and coordination of care during this critical transition period.

Given the benefits of structured GH approaches and the observed variability in local stabilization practices, implementing a standardized GH protocol was expected to reduce heterogeneity and improve key clinical parameters. Grounded in evidence-based guidelines and principles of process standardization and teamwork, this quality improvement initiative aimed to enhance anticipation, communication, and task sequencing, thereby improving procedural efficiency and consistency of care.

The primary objective was to assess its impact on process efficiency and on early clinical outcomes, including admission temperature and blood glucose levels. A secondary objective was to evaluate staff perceptions of the protocol, to understand its impact on workflow, teamwork, and perceived care quality, and to identify factors supporting its sustainable implementation.

## 2 Methods

### 2.1 Context

This quality-improvement (QI) project was conducted in the neonatal intensive care unit (NICU) of the University Hospital Center of Liège (Belgium), a 25-bed university-affiliated level IIIb unit. Approximately 450 infants are admitted each year, of whom 20% are born before 31 weeks' gestation (wGA). At project initiation, variability existed in delivery-room stabilization workflows and early NICU admission processes for very preterm infants (<32 wGA)—particularly regarding task sequencing, timing of umbilical venous catheter (UVC) insertion, thermoregulation, and early glucose assessment. These observations highlighted the need for a standardized and coordinated approach during the first hour of care.

Eligibility criteria for GH protocol implementation and study inclusion were gestational age <31 wGA and/or estimated fetal weight (EFW) <1,300 g, consistent with local practice for systematic UVC insertion. Exclusion criteria were outborn infants, major congenital anomalies, and lack of parental consent for data analysis.

### 2.2 Interventions

#### 2.2.1 Intervention design and implementation

In 2021, a multidisciplinary team (neonatologists, NICU nurses, and pediatric residents) designed a structured GH protocol to standardize resuscitation, stabilization, and early admission of preterm infants. A review of literature (5) helped to identify key components of effective early stabilization and to ensure alignment with current guidelines. To support implementation, staff received structured in-person multidisciplinary training through educational sessions combining theoretical presentations and simulation-based practice, as well as role-specific flowcharts and visual reminders in the delivery room (DR). The protocol was pilot tested and refined over four months before study launch to ensure feasibility and integration into routine practice.

#### 2.2.2 Delivery-room preparation and anticipation

Before each birth, a predefined team was assembled, including a neonatologist, two NICU nurses (one primarlily responsible for DR care and one for NICU admission), and a resident. When available, an additional staff member handled real-time documentation. For each team member, a flowchart outlined specific roles and responsibilities (Table 1). Additionally, a poster reminder was displayed in the DR resuscitation area, and individual memory cards were available.

**Table 1:** Flowchart detailing specific roles and responsibilities for each team member (TPR, T-piece resuscitator; m, minute of life; CPAP, continuous positive airway pressure; DR, delivery room; NICU, neonatal intensive care unit; UVC, umbilical venous catheterization.

**Timeframe**		**Senior—Neonatologist**	**Resident**	**Nurse 1**	**Nurse 2**
ANTENATAL	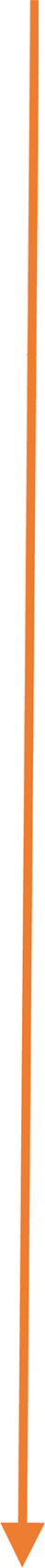	Inform parents Involve parents in patients care Check obstetrical records Remind obstetrician on delayed cord clamping + timing	Check obstetrical records Check equipment in DR: TPR, suction, intubation…	Prepare heated and humidified incubatorPrepare heated mattress (39 °C) Check equipment DR: resuscitation cart, polyethylene bag, hat, … Prepare transport system in DR	Prepare NICU room + check equipmen Prepare standard parenteral nutrition, caffeine and vitamin Prepare equipment for UVC placemen Prepare lab tubes
DELIVERY ROOM M0		Transition support according to resuscitation algorithm: TPR, ventilation, oxygen titration Thermal protection	Set up ventilatory support (TPR) and stabilize breathingThermal protection: polyethylene bag	Start timer Place SpO2 sensor on the right hand Place ECG electrodes on the back Place Bahr clamp	Available for additional help in DR
M10		Consider transfer Presentation to the coparent	Prepare UVC placement	Take head circumference (+/- height) and fit CPAP cap	
ADMISSION NICU M15		Check ventilatory support settings and effectiveness		Install child in NICU room (4-handed) Remove polyethylene bag, dry newborn and place on heated mattress Insert nasogastric and thermal probes	Install child in NICU room (4-handed) Weigh Preparation for UVC placement: cleaning of periumbilical area with soap + disinfection with chlorhexidine solution, help with sterile gowning and table preparation
M20		Setting up central lines Check UVC position on x-ray	Setting up central lines Draw blood for analysis	Child support and contention during UVC placement Start PN as soon as possible (before fixation of UVC) + caffeine	Creation of medical record Assist x-ray to check UVC position Collect lab tubes and perform blood gazes
M45		Prescription in medical record Assess need for surfactant		Reposition child into a cocoon Consider removing heated mattress depending on temperature Take blood pressure, consider NIRS Adjust parental feeding rate Administration of vitamin K, ophthalmic disinfection ± antibiotics	Help with repositioning of the child Prepare antibiotics
M60		Inform parents Assess need for surfactant		Closing incubator, dimming the lights and install incubator cover	
M120			Retrieve laboratory results Complete medical file	Retrieve laboratory results Complete medical file Notify neonatologist if FiO_2_ >25% in <26 wGA or >30% in >26 wGA: surfactant?	Minimal touch Notify neonatologist if FiO_2_ >25% in<26 wGA or >30% in >26 wGA: surfactant?

When a preterm birth was anticipated, the neonatologist reviewed obstetric records and subsequently counseled the parents. DR equipment was prepared and checked, including the resuscitation cart, thermoregulation supplies (DR prewarming, radiant warmer preheating, polyethylene bag, hat, heated mattress), and respiratory support equipment (T-piece resuscitator, intubation equipment, oxygen blender, surfactant). The transport incubator was preheated and positioned near the DR.

Simultaneously, NICU admission was anticipated: bedside equipment (IV pumps, CPAP or ventilator) was checked, the UVC insertion kit prepared, and parental nutrition (PN), caffeine, vitamin K and antibiotics readied according to clinical context.

Initial DR stabilization followed the 2021 European Resuscitation Council guidelines (6), using a T-piece resuscitator for early CPAP or mask ventilation with oxygen titration starting at 21%; and at 30% for <28wGA.

#### 2.2.3 NICU admission process

Once stabilized, infants were transferred in a preheated humidified incubator (34 °C, 85%). Thermal protection measures were maintained throughout the admission with continuous temperature monitoring. Initial weighing allowed medication and infusion prescriptions, after which UVC insertion was initiated promptly. Incubator openings were minimized until UVC placement, and radiant heating with a prewarmed heated mattress was used. Fluids were initiated before x-ray confirmation of central line placement. Caffeine, vitamin K and antibiotics were administered as early as possible. After successful UVC placement, the incubator was closed, blood pressure measured, and stimulation minimized for the following two hours. The unit had a rescue surfactant policy primarily using less invasive surfactant administration (LISA), according to European guidelines thresholds (7).

A dedicated real-time admission record supported data collection (Supplementary Figure S1).

### 2.3 Study of the intervention

A prospective observational study was conducted from January 1, 2022 to July 31, 2023. As a comparative arm, all eligible infants born in 2019 were retrospectively included to avoid bias related to organizational changes during the COVID-19 epidemic. All thermoregulation and stabilization devices were available in both groups.

### 2.4 Measures

#### 2.4.1 Outcomes and process measures

Procedural and patient-oriented outcomes were predefined. Time to incubator closure, used as a proxy for admission completion, was the main procedural outcome, as it allows assessment of the efficiency of the admission process. Mortality, hypoglycemia (glycemia <47 mg/dL measured by point-of-care analyzer or glucose reader during venous access insertion and within the first 24 h), and thermal stability (temperature at admission and at procedure completion; hypothermia defined as rectal temperature <36.5 °C and hyperthermia >38 °C) were the main clinical outcomes.

Time to initiation of PN, caffeine and antibiotics were selected as secondary procedural outcomes. Markers of cardiorespiratory stability during the procedure–including hypotension (mean arterial pressure below gestational age in mmHg), bradycardia (heart rate <100/min), endotracheal intubation, duration of mechanical ventilation, and surfactant administration–were assessed as secondary clinical outcomes.

Major comorbidities of prematurity were also assessed: moderate-to-severe bronchopulmonary dysplasia (BPD) according to the 2001 NIH definition (8), patent ductus arteriosus requiring medical or surgical therapy (PDA), necrotizing enterocolitis (NEC) (Bell stage 2 or 3), severe intraventricular hemorrhage (Papille's grade 3 or 4, sIVH), cystic periventricular leukomalacia (cPVL), severe retinopathy of prematurity (sROP) (defined as stage ≥3), proven early- (EOS) and late-onset sepsis (LOS).

#### 2.4.2 Assessment of contextual influences

Contextual factors including staffing patterns, DR workload, and on-call periods were qualitatively tracked through team discussions during protocol roll-in and implementation. These contextual elements were analyzed to understand contributors to variations in protocol adherence and intervention timeliness.

### 2.5 Statistical analysis

Data analysis was performed using R® statistical software. Numerical and graphical checks were conducted to ensure database reliability. Student's t-test was used to compare normally distributed continuous variables, whereas the Mann-Withney test was applied otherwise. Chi-square and Fisher tests were applied to qualitative variables. The significance threshold was set at *p* < 0.05.

### 2.6 Ethical considerations

The project was approved by the local ethics committee on December 21, 2021, and registered in ClinicalTrials.gov (NCT05175911). The study was conducted as part of a structured QI initiative. Written parental consent for inclusion in the analysis was obtained after admission. The study followed the SQUIRE 2.0 guidelines for reporting quality-improvement studies (9).

## 3 Results

Between January 2022 and July 2023, 94 infants met inclusion criteria. The GH protocol was not implemented in 17 cases, mainly due to organizational constraints such as limited staff availability during on-call hours or the inability to anticipate the birth in situations of vital emergency. During the initial phase of the project, 11 infants were not included as the protocol was still being introduced and integrated into routine clinical practice. Parental refusal accounted for three additional exclusions. Ultimately, 77 Infants were included in the GH cohort. As a comparative arm, we retrospectively reviewed all 72 patients meeting the same inclusion criteria who were born in 2019.

### 3.1 Demographics

Both GH and control groups were comparable for gestational age [28.8(27.5–30.3) vs. 28.9(26.7–30.5)wGA -*p* = 0.75], birth weight [1100(850–1,210) vs. 1,125(879–1,370)g -*p* = 0.38], gender, Apgar score at 5 min, and prenatal steroid exposure. The proportion of small for gestational age (SGA) infants was higher in the GH group (32.5 vs. 9.7% -*p* < 0.001), and more infants were delivered by caesarean section (87 vs. 67% -*p* = 0.006). The median time to cord clamping was 30 (19.75–36.75) seconds in the GH group, while corresponding data were not available for the control group. Patients' demographics are described in Table 2.

**Table 2:** Patients' demographics [median (IQR)—*n*(%)].

Variables	Golden Hour (*n* = 77)	Controls (*n* = 72)	*P-*value
Gestational age (weeks)	28.8 (27.5–30.3)	28.9 (26.7–30.5)	0.75
Birth weight (grams)	1,100 (850–1,210)	1,125 (879–1,370)	0.38
Sex: male	38 (49%)	41 (57%)	0.44
Small for gestational age	25 (32.5%)	7 (9.7%)	**<0.001**
Complete antenatal steroids exposure	60 (78%)	52 (72%)	0.54
C-section	67 (87%)	48 (67%)	**0**.**006**
Out-of-hours birth	35 (45%)	40 (55%)	0.28
Apgar at 5 min	9 (7–9)	8 (7–9)	0.25
Mask ventilation in Delivery room	59 (77)	58 (80)	0,7
Intubation in Delivery room	5 (6.4)	10 (14)	0.22

Statistically significant *p*-values are shown in bold.

### 3.2 Procedural outcomes

**Figure 1:**
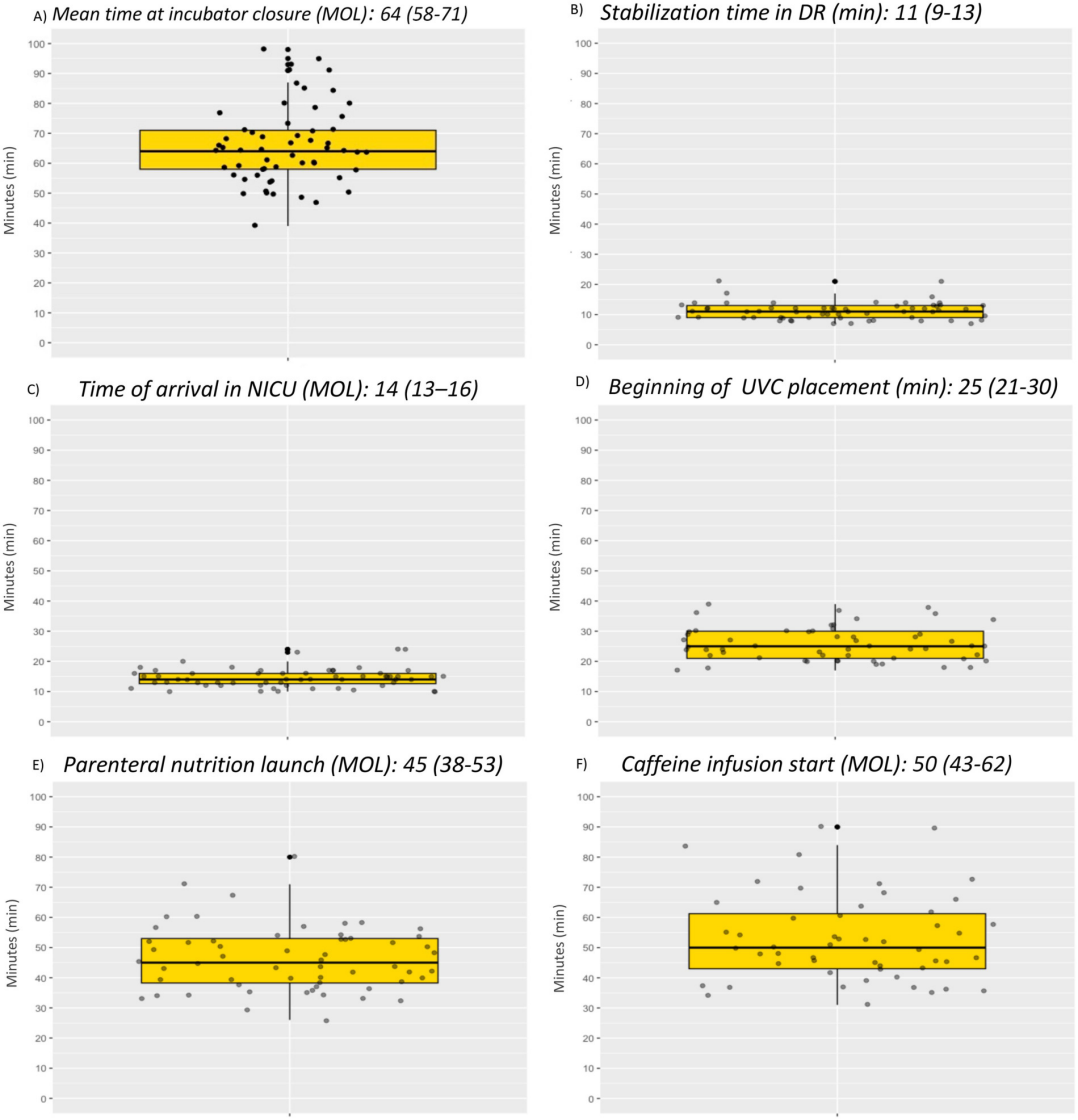
Procedural outcomes in GH cohort. min, minutes; DR, delivery room; MOL, Minutes of life; NICU, neonatal intensive care unit; UVC, umbilical venous catheterization.

End of procedure, defined as incubator closure within one hour, was achieved in 40% of GH admissions, with a median time of 64 (58–71) MOL. Average DR stabilization time was 11(9–13)MOL, with arrival in the NICU at 14 (13–16)MOL (Figure 1).

UVC insertion began at 25(21–30)MOL, allowing early initiation of PN at 45(38–53)MOL (Figure 1). Because UVC placement coincided with the first blood glucose sampling, the timing of this measurement directly reflected successful catheter insertion. The initial blood glucose was measured significantly earlier after implementation of the GH protocol [43(35–50) vs. 63(53–77)MOL -*p* < 0.001].

No retrospective comparative data were available for other procedural outcomes.

### 3.3 Clinical outcomes

Mortality rates were similar in both groups (3.9 GH vs. 8.3% controls -*p* = 0.46). Hypoglycemia incidence did not differ significantly (44 vs. 39% -*p* = 0.63), nor did initial blood glucose values [44(30–60) vs. 44(27–57)mg/dL -*p* = 0.86]. Hypothermia on admission decreased significantly in the GH group (31.5 vs. 50% -*p* = 0.03), while median admission temperatures were comparable (36.6 vs. 36.4 °C -*p* = 0.41). Hyperthermia was observed in 4 GH infants vs. 1 control (*p* = 0.38).

Rates of major prematurity related comorbidities (BPD, NEC, sIVH, cPVL, sROP) were similar between groups. Fewer cases of PDA were treated in the GH group (14.2 vs. 31.4% -*p* = 0.02). Trends toward earlier implementation of LISA and reduced intubation were not statistically significant (*p* = 0.1 and *p* = 0.12, respectively) (Table 3).

**Table 3:** Clinical outcomes [median (IQR)—*n*(%)].

Variables	Golden Hour (*n* = 77)	Controls (*n* = 72)	*P-*value
Admission temperature ( °C)	36.6 (36.2–36.9)	36.4 (36–36.9)	0.41
Hypothermia on admission	23 (31.5%)	36 (50%)	**0**.**03**
Hyperthermia on admission	4	1	0.38
Hyperthermia at the end of procedure	1 (*n* = 43)	0 (*n* = 4)	NA
First glycemia (mg/dL)	44 (30–60)	44 (27–57)	0.86
Hypoglycemia on admission	41 (53%)	36 (50%)	0.82
Severe hypoglycemia on admission (<25 mg/dL)	12 (16%)	16 (22%)	0.41
Time to first glycemia measure (min)	43 (35–50)	63 (53–77)	**< 0.001**
BPD moderated/severe	13 (17.5%)	10 (15.1%)	0.88
Intubation rate	15 (19.5%)	23 (31.9%)	0.12
Time to surfactant administration (min)	164 (120–690)	275 (120–840)	0.24
LISA before 2h	12 (*n* = 28)	5 (*n* = 27)	0.1
Red cell Transfusion (one or more)	25 (33%)	25 (35%)	0.95
PDA treatment	11 (14.2%)	22 (31.4%)	**0**.**02**
PDA surgical	0	1	0.12
Early-onset sepsis	4 (5.2%)	0	0.14
Late-onset sepsis	20 (27%)	15 (21%)	0.52
NEC	4 (5.2%)	2 (2.8%)	0.74
sROP	0	0	1
sIVH	2 (2.6%)	3 (4.1%)	0.94
cPVL	0	1	0.96
Death	3 (3.9%)	6 (8.3%)	0.46

min, minutes; BPD, bronchopulmonary dysplasia; LISA, less invasive surfactant administration; PDA, patent ductus arteriosus; NEC, necrotizing enterocolitis; sROP, severe retinopathy of prematurity; sIVH, severe intraventricular hemorrhage; cPVL, cystic periventricular leukomalacia.

### 3.4 Subanalysis for extremely preterm infants

The subgroups including infants born at < 27 wGA or with a birth weight <850 g were similar in size in each group (23 vs. 22 infants), but showed a significant difference in SGA proportion (*p* = 0,003), resulting in a less immature population in GH arm (27,18 vs. 25,6 wGA—*p* = 0,002) (Supplementary Table S2). We also observed a significant difference in cesarean incidence (*p* < 0,001).

Glycemia at admission and the occurrence of hypoglycemia were similar (*p* = 0,91), while the shorter time to perfusion was maintained (41,5 vs. 62,5 min—*p* < 0,001). No differences were observed in hypothermia occurrence or median temperature at admission (Supplementary Table S3).

No significant differences were found in the common comorbidities of prematurity, excepted for a fewer PDA requiring treatment (*p* = 0,006).

### 3.5 Neonatal care team perception and contextual factors

The perception of the newly implemented GH protocol within the neonatal care team was assessed through an online survey (Supplementary Table S4). We did not have comparative before/after data. Characteristics of staff members who took part in the survey are described in supplemental files (Supplementary Tables S5 and S6).

Overall, the implementation of the GH protocol was positively received by all team members, leading to a reduction in stress expressed by the staff (78,5%) and a perception of improved quality of work (72,3%). Key components that led to these positive results were clear role assignments (92,8%) with improved communication (90,5%) and better anticipation facilitating neonatal care. Visuals aids (such as cards, protocol, powerpoint, etc.) were deemed useful to improve understanding of each team member's role, and conducive to smooth running of the process (76,1%).

## 4 Discussion

This study evaluated the implementation of a systematic approach for managing preterm infants born at <31 wGA and/or with EFW <1,300 g during their first hour of life. This approach was based on encouraging data from the literature regarding GH protocols (5), in order to improve neonatal outcomes.

Although the target of completing admissions within 60 MOL was not consistently achieved, staff feedback suggests that the GH protocol has positively impacted the organization of care, with notable improvements in anticipation, communication, and teamwork. The average incubator closure time 64 MOL was close to the predefined target and could likely improve with further practice. One potential explanation is the unit strong focus on training residents, who tend to require longer times for UVC insertion.

Primary clinical objectives of GH procedures in neonatology include early administration PN administration to promote glycemic stability and maintenance of normothermia throughout the management process.

Hypoglycemia is a common and serious complication in preterm infants, particularly in the immediate postnatal period, as it may lead to seizures and brain injury (10) if untreated. These infants are especially vulnerable due to limited glycogen stores, reduced fat reserves, and immature gluconeogenic capacity. The abrupt interruption of the placental glucose supply at birth leads to a physiological drop in plasma glucose levels, typically reaching a nadir within the first two hours of life. Although there is no universal definition of neonatal hypoglycemia, the American Academy of Pediatrics (AAP) has set a plasma glucose threshold of 47 mg/dl to define hypoglycemia in neonates (11). Given the increased vulnerability, close monitoring and prompt management are essential to prevent adverse neurological outcomes.

No significant difference in hypoglycemia at admission was observed between GH and control groups, although the GH cohort included a higher proportion of SGA infants, limiting comparability. The evaluation of early metabolic management showed a significantly shorter time to first blood glucose measurement in the GH group, consistent with earlier umbilical venous line placement and faster initiation of initial care. Because data on PN initiation were unavailable in the control cohort, this parameter could not be compared. Overall, hypoglycemia rates remained low in both groups, but earlier glycemic assessment observed in the GH cohort suggests improved efficiency in early metabolic management.

Currently, there is no clearly established concensus regarding the use of peripheral versus umbilical venous access at birth, particularly with respect to early glycemia. However, several studies support umbilical venous catheterization in preterm infants (12). In our practice, we opted for umbilical venous catheterization, as it enables improved delivery of PN and provides more reliable longer-term intravenous access, while still allowing for early initiation of PN.

According to the World Health Organization (WHO), normothermia is defined as a temperature between 36.5 °C and 37.5 °C (13). Preterm and very low birth weight infants are particularly vulnerable to hypothermia given their higher surface area-to-body mass ratio, reduced subcutaneous fat, limited metabolic reserves, and immature thermoregulation. Adequate thermal protection is therefore critical for the initial stabilization of newborns. Numerous studies described correlations between hypothermia and increased mortality (14–17), IVH (15–17), NEC (15)^]^ and LOS (14, 15). Many strategies help to improve temperature: use of polyethylene bags (18, 19), caps (18–20), pre-warming of DR (20), thermal mattress or pre-heated radiant warmers (21), heated humidified gas (18–20) and heated humidified incubators.

In our study, hypothermia significantly decreased following GH protocol implementation (50% to 30%). These findings are consistent with recent meta-analysis, where hypothermia decreased from 61% to 30%. Despite the small sample size, this result remained clinically meaningful and statistically significant. However, the higher proportion of SGA infants and the increased rate of cesarean section in the GH group may have influenced thermoregulation outcomes. In a meta-analysis, this mode of delivery was associated with a higher risk of hypothermia (22). Potential explanations include colder operating room temperature, as well as exposure to narcotic drugs that may affect the brain's thermoregulatory center (22). These factors may further support the significance of our findings. Finally, careful monitoring remains essential to avoid hyperthermia and ensure optimal thermal management.

Although previous studies suggest a trend toward reduced BPD following GH protocol implementation (7, 23), no significant decrease was observed in our cohort, likely due to limited statistical power and a higher mean gestational age. In our unit, LISA is systematically performed under analgesia; therefore, earlier UVC placement allowed for timely analgesia and surfactant delivery, enhancing both procedural efficiency and patient comfort. Nevertheless, this requirement for analgesia may slightly delay surfactant administration compared with centers performing LISA without prior analgesia, highlighting a trade-off between rapid intervention and optimal pain management. These findings underscore the importance of balancing procedural timing with patient-centered care.

The reduced need for medical PDA treatment after GH implementation likely reflects broader changes in clinical practice over time rather than a direct protocol effect. As this was an observational study, all outcomes should therefore be interpreted as associations rather than causal effects.

This study also aimed to evaluate team perceptions of the GH protocol. While we were unable to demonstrate significant improvements in clinical outcomes other than thermoregulation—possibly due to the lack of major changes in overall care—the GH protocol has positively impacted team dynamics. Notable improvements in anticipation, communication, and teamwork have enhanced team perception and overall job satisfaction.

The management of a newborn during the GH is a complex multidisciplinary process requiring efficient organization. Effective teamwork is essential, and it is strengthened through structured simulations, where clear roles and responsibilities are defined for each team member (24–27). Such approaches improve communication and reduce the perceived complexity of admission procedure. By establishing well-defined team roles and implementing GH protocols, improvements were observed in staff efficiency, overall organization, and potentially patient outcomes. In our NICU, visual aids were introduced to clarify team responsibilities and further improve coordination (27).

Although overall team perception was positive, a proportion of staff reported increased stress, limited familiarity with the protocol, or no improvement in work satisfaction. These responses may reflect the initial implementation phase, during which staff were closely observed and timed, potentially increasing performance pressure. In addition, lower familiarity with the protocol was mainly reported by less experienced staff or those less frequently involved in delivery room care. These findings likely illustrate the learning curve and adaptation period inherent to quality improvement initiatives.

This study has several limitations. First, its monocentric design may limit the generalizability of the findings in other settings with different patient populations or care practices. Second, the relatively small sample size may have reduced the statistical power to detect differences between groups. In addition, the study population consisted predominantly of relatively more mature preterm infants (median 28.8wGA, median BW∼1,100 g). In Belgium, active management below 24wGA was not routinely performed. However, our sub-analysis of extremely preterm infants confirmed a shorter time to perfusion and the safety of GH protocols, although the GH group was relatively more mature. Further studies are needed to better evaluate GH protocols in this high-risk group, in whom such structured approaches may be particularly relevant despite greater clinical instability.

The retrospective 2019 cohort posed also methodological challenges, as data were paper-based, increasing the risk of inaccuracies—particularly in time recording—and some variables were inconsistently documented or not directly comparable with the prospective cohort. Finally, the retrospective design meant that some variables were inconsistently documented or not directly comparable between groups.

## 5 Conclusion

The GH protocol was associated with modest improvements in thermoregulation and organizational efficiency, while most clinical outcomes remained unchanged. Nevertheless, improved teamwork and positive staff perception highlight its potential benefits. Some staff reported increased stress or limited familiarity with the protocol, highlighting the importance of training and adaptation during implementation. Further refinement and larger multicentric studies are needed to fully evaluate its impact on neonatal outcomes.

## Data Availability

The raw data supporting the conclusions of this article will be made available by the authors, without undue reservation.
