# Supplementary material for: Implementation of a Golden Hour protocol for initial management of preterm infants: a quality improvement study
Source: Front Pediatr. 2026 May 20;14:1832031. doi: 10.3389/fped.2026.1832031 (PMC13230198; doi:10.3389/fped.2026.1832031)
Supplement: Supplementary file 1 [file Supplementaryfile1.docx]

1. **SUPPLEMENTAL FILES:**

**eFig 1:** Neonatal admission record

**eTable 2:** Patients’ demographics in extremely preterm infants (median (IQR) – n(%))

**eTable 3:** Clinical outcomes in extremely preterm infants (median (IQR) – n(%))

**eTable 4** : Neonatal care team perception survey

**eTable 5:** Characteristics of staff members participating in the survey - Experience

**eTable 6:** Characteristics of staff members participating in the survey – Team member’s role/profession

**eFig 1:** Neonatal admission record

GA : …………… wGA Hour of birth : ………h…..…

Prenatal :

- Maturation cure (Celestone©):  0 – 1 – 2 – 3 doses

- Neuroprotection by Mg sulfate: YES-NO

- Ultrasound monitoring: normal - ………………………………………………

- Twin pregnancy ?: NO - …………………………………………………..

Delivery :

- Vaginal – C-section /  iterative – semi-urgent – urgent

Birth :

- Delayed cord clamping:  YES – NO  (duration : ……………..sec)  - Milking : YES – NO

- Thermal protection: Polyethylene bag at ………………….. min - DR temperature: ………………°C

- Ventilatory support:

- Ventilation duration: ……………………min
- FiO_2_ :   max : ………………% - 5’ : ………………% - 10’ : ………………%
- Intubation: YES – NO      -   Surfactant : YES – NO      - LISA : YES – NO

- Parameters taken (HC et height) : YES – NO    Weight : …………… g    Height : ……………. cm   HC : …………… cm

- Transfer decision at ………h……… (M………) -  In the incubator at ……….h……… (M……….)

At admission :

- Time of arrival: ………h……… (M………)

- First temperature (rectal probe): ………………°C

- Nasogastric tube before UVC: YES – NO   - reason: ………………………………………………………………………………………………

- Back electrode placement: YES – NO - reliability ? ……………………………………………………………………………

- Start of UVC placement at ………h……… (M………)

- First glycemia ? ……………… mg/dl at ………h……… (M………)

- UAC placement ? YES – NO - in place at ………h……… (M………)

- Start of parenteral nutrition at ………h……… (M………)

- Administration of caffeine at ………h……… (M………)

- UVC field withdrawal at ………h……… (M………)

- Closing the incubator at ………h……… (M………) – Temperature at closing : ………………°C

Clinical tolerance:

- Evolution of FiO2 during UVC placement: ………………………………………………………………………………………………

- Bradycardia ? Tachycardia ?………………………………………………………………………………………………………………………

- First BP ? ………………mmHg at ………h………

Evolution:

- LISA ? YES – NO  at ……….h……… (M………) - Intubation ? YES – NO at ………h………

- Glycemic evolution: ………………………………………………………………………………………………………………………………………

- Antibiotics: YES – NO at ………h………

- First alimentation at ………h………

- Concordance of parameters (HC et height) checked after a few days: YES – NO

Golden Hour :

- Number of members ? ……… Nurses - ……… Residents - ……… Supervisor

- Team perception : ………………………………………………………………………………………………………………………………………………

- Time exceeded ? Reason ? ………………………………………………………………………………………………………………………………

- Stopped ? Reason ? ………………………………………………………………………………………………………………………………………………

- Exclusion ? Reason ? ……………………………………………………………………………………………………………………………………………

Last name:

First name:

Birth date:

**eTable 2:** Patients’ demographics in extremely preterm infants (median (IQR) – n(%))

| **Variables** | **Golden Hour**  **(n = 23)** | **Controls**  **(n = 22)** | ***P-*value** |
| --- | --- | --- | --- |
| Gestational age (weeks) | 27.2 (26.1-28.4) | 25.9 (24.8-26.5) | **0.002** |
| Birth weight (grams) | 747 (672-822) | 759 (677-804) | 0.799 |
| Sex: male | 8 (35%) | 18 (82%) | **0.004** |
| Small for gestational age | 11 (47.8%) | 1 (4.5%) | **0.003** |
| Complete antenatal steroids exposure | 23 (100%) | 16 (73%) | **0.024** |
| C-section | 23 (100%) | 12 (54%) | **<0.001** |
| Out-of-hours birth | 11 (48%) | 15 (68%) | 0.28 |
| Apgar at 5 min | 9 (8-9) | 8 (6,2-8) | 0.10 |
| PPV in DR | 19 (83) | 22 (100) | 0.13 |
| Intubation in DR | 3 (13) | 6 (27) | 0.41 |

**eTable 3:** Clinical outcomes in extremely preterm infants (median (IQR) – n(%))

| **Variables** | **Golden Hour**  **(n = 23)** | **Controls**  **(n = 22)** | ***P-*value** |
| --- | --- | --- | --- |
| Admission temperature (°C) | 36.65 (36.1-36.8) | 36.35 (36-37) | 0.91 |
| Hypothermia on admission | 8 (40%) | 12 (55%) | 0.53 |
| First glycemia (mg/dl) | 51 (34.5-69.5) | 47 (41.2-62.5) | 0.81 |
| Hypoglycemia on admission | 8 (35%) | 9 (41%) | 0.91 |
| Severe hypoglycemia on admission (<25 mg/dl) | 4 (17%) | 3 (14%) | 1 |
| Time to first glycemia measure (min) | 41.5 (32-47) | 62.5 (58.5-76.5) | **< 0.001** |
| BPD moderated/severe | 8 (42%) (n=19) | 6 (35%) (n=17) | 0.94 |
| Intubation rate | 9 (39%) | 15 (38%) | 0.10 |
| Time to surfactant administration (min) | 140 (96-210) | 180 (120-1200) | 0.45 |
| PDA treatment | 7 (30%) | 16 (76%) | **0.006** |
| PDA surgical | 0 | 1 | 0.3 |
| NEC severe | 1 (4%) | 3 (14%) | 0.57 |
| sROP | 1 | 0 | 1 |
| sIVH | 1 (4%) | 3 (14%) | 0.57 |
| cPVL | 0 | 0 | / |
| Death | 3 (13%) | 6 (27%) | 0.41 |

***Abbreviations:*** *min=minutes; BPD=bronchopulmonary dysplasia; LISA=less invasive surfactant administration; PDA=patent ductus arteriosus; NEC=necrotizing enterocolitis; sROP=severe retinopathy of prematurity; sIVH=severe intraventricular hemorrhage; cPVL=cystic periventricular leukomalacia*

**eTable 4:** Neonatal care team perception survey

|  | **SATISFACTION RATING SCALE** | | | |  | |  |
| --- | --- | --- | --- | --- | --- | --- | --- |
|  | | **Completely disagree** | **Disagree** | **Agree** | | **Completely agree** | |
| The roles of each team member are clearly established and respected (n=42) | | 1 (2.4%) | 2 (4.8%) | 24 (57.1%) | | 15 (35.7%) | |
| Each team member shares clear information’s to enable rapid decision-making (n=42) | | 0 | 4 (9.5%) | 30 (71.4%) | | 8 (19%) | |
| I encounter difficulties in carrying out the procedure (n=41) | | 6 (14.6%) | 18 (43.9%) | 17 (41.5%) | | 0 | |
| Information about patient care is clearly explained to parents, and fathers are involved in the child’s care (n=41) | | 0 | 10 (24.4%) | 26 (63.4%) | | 5 (12.2%) | |
| Golden Hour protocol enables better organization of patient care (n=42) | | 0 | 3 (7.1%) | 23 (54.8%) | | 16 (38.1%) | |
| Anticipation and preparation of the Golden Hour protocol reduces the level of stress felt by the team (n=42) | | 1 (2.4%) | 8 (19%) | 25 (59.5%) | | 8 (19%) | |
| Golden Hour protocol improves satisfaction with quality of work (n=41) | | 1 (2.4%) | 8 (19.5%) | 24 (52.8%) | | 8 (19.5%) | |
| The Golden Hour protocol is applied in accordance with NIDCAP (n=42) | | 2 (4.8%) | 16 (38.1%) | 21 (50%) | | 3 (7.1%) | |
| If no agreement: The Golden Hour protocol could be applied in accordance with NIDCAP principles (n=24) | | 0 | 0 | 17 (70.8%) | | 7 (29.2%) | |
| I have a good knowledge of the procedure (n=42) | | 1 (2.4%) | 10 (23.8%) | 29 (69%) | | 2 (4.8%) | |
| I know what is expected of me during the procedure (n=42) | | 1 (2.4%) | 3 (7.1%) | 30 (71.4%) | | 8 (19%) | |
| I know that “Golden Hour” visual aids (cards, protocol, powerpoint, etc.) are available in the department (n=42) | | 2 (4.8%) | 8 (19%) | 24 (57.1%) | | 8 (19%) | |
| I try to consult the Golden Hour procedure or visual aids before applying it (n=41) | | 1 (2.4%) | 10 (24.4%) | 21 (51.2%) | | 9 (22%) | |
| The “Golden Hour” visual aids (card, protocol, powerpoint,etc.) enable a better understanding of the role of each team member and are conducive to the smooth running of the treatment (n=40) | | 0 | 2 (5%) | 22 (55%) | | 16 (40%) | |
| The principles of anticipation and preparation for the “Golden Hour” have become a departmental habit (preparation of infusions, bed location, equipment, prescriptions) (n=42) | | 0 | 4 (9.5%) | 24 (57.1%) | | 14 (33.3%) | |

**eTable 5:** Characteristics of staff members participating in the survey – Experience

| **Number of years in the department** | **Number (%)** |
| --- | --- |
| < 2 years | 10 (23.8%) |
| 2-5 years | 8 (19%) |
| 5-10 years | 4 (9.5%) |
| 10-15 years | 3 (3.7%) |
| 15-20 years | 2 (4.8%) |
| > 20 years | 15 (35.7%) |

**eTable 6:** Characteristics of staff members participating in the survey – Team member’s role/profession

| **Profession** | **Number (%)** |
| --- | --- |
| Nurse with admission and DR experience | 23 (54.8%) |
| Nurse without DR experience | 6 (14.3%) |
| Neonatologist | 8 (19%) |
| Pediatric resident | 5 (11.9%) |
